# Biopolymer Compositions Based on Poly(3-hydroxybutyrate) and Linear Polyurethanes with Aromatic Rings—Preparation and Properties Evaluation

**DOI:** 10.3390/polym16121618

**Published:** 2024-06-07

**Authors:** Beata Krzykowska, Anna Czerniecka-Kubicka, Anita Białkowska, Mohamed Bakar, Miroslava Kovářová, Vladimir Sedlařík, Dominika Hanusova, Iwona Zarzyka

**Affiliations:** 1 Department of Organic Chemistry, Faculty of Chemistry, Rzeszów University of Technology, Powstańców Warszawy 6, 35-959 Rzeszów, Poland; 2 Department of Experimental and Clinical Pharmacology, Medical College of Rzeszow University, The University of Rzeszow, 35-310 Rzeszów, Poland; aczerniecka@ur.edu.pl; 3 Faculty of Applied Chemistry, Radom University, Chrobrego 27, 26-600 Radom, Poland; a.bialkowska@uthrad.pl (A.B.); m.bakar@wp.pl (M.B.); 4 Centre of Polymer Systems, University Institute, Tomas Bata University in Zlin, Tr. T. Bati 5678, 76001 Zlin, Czech Republic; kovarova@utb.cz (M.K.); sedlarik@utb.cz (V.S.); d_hanusova@utb.cz (D.H.)

**Keywords:** polyester, polyurethane, structure–properties relationship, mechanical properties, thermal properties

## Abstract

Polymer biocompositions of poly(3-hydroxybutyrate) (P3HB) and linear polyurethanes (PU) with aromatic rings were produced by melt-blending at different P3HB/PU weight ratios (100/0, 95/5, 90/10, and 85/15). Polyurethanes have been prepared with 4,4′-diphenylmethane diisocyanate and polyethylene glycols with molar masses of 400 g/mol (PU400), 1000g/mol (PU1000), and 1500 g/mol (PU1500). The compatibility and morphology of the obtained polymer blends were determined by infrared spectroscopy (FTIR), scanning electron microscopy (SEM), and differential scanning calorimetry (DSC). The effect of the polyurethane content in the biocompositions on their thermal stability and mechanical properties was investigated and compared with those of the native P3HB. It was shown that increasing the PU content in P3HB-PU compositions to 10 wt.% leads to an improvement in the mentioned properties. The obtained results demonstrated that the thermal stability and mechanical properties of P3HB were improved, particularly in terms of increasing the degradation temperature, reducing hardness, and increasing impact strength. The best thermal and mechanical properties were shown by the P3HB-PU polymer compositions containing 10 wt.% of polyurethane modifiers, especially PU1000, which was also confirmed by the morphology analysis of these biocompositions. The presence of polyurethanes in the resulting polymer biocomposites decreases their glass transition temperatures, i.e., makes the materials more flexible. The resulting polymer biocompositions have suitable mechanical properties and thermal properties within the processing conditions for the predicted application as biodegradable, short-lived products for agriculture.

## 1. Introduction

Drastic climate change, rapid depletion of natural resources, and increased greenhouse-gas emissions are just a few of the consequences of continued human interference with the environment. Among the most notable is the significant pollution of the environment by waste from everyday subjects. On the other hand, the global production of plastics has increased drastically over the past few years, which has been controversial. The European Commission reports that Europe alone produces 26 million tons of plastic waste annually [1]. Due to its inability to biodegrade and the lack of developed appropriate disposal methods, the waste is dumped in landfills or, in the worst case, incinerated. Toxic products of their partial decomposition under the influence of atmospheric factors as well as disposal processes are released into the environment, which poses a threat to the health of all living organisms [2]. In addition, oil deposits are non-renewable. It is estimated that, with their depletion, the cost of extraction will increase significantly and, thus, their price. Therefore, the scientific community continues to focus on solutions that address the reduction of synthetic polymers’ effects [3].

Solutions are constantly being sought to meet the challenges of reducing plastics derived from fossil fuels and petroleum. A potential solution is the use of products derived from biomass, as well as increasing the share of biodegradable biopolymers in materials [4,5]. Biopolymers are competitive not only in terms of their functional properties but also in terms of cost. Most biopolymers are biodegradable [6,7] and are generally biocompatible and non-toxic to the environment [8]. However, despite their many advantages, they also have a lot of disadvantages for applications in structural materials. However, they can be slightly improved or modified, making them more accessible. To increase the biodegradability of some materials or to partially reduce reliance on artificial materials, they are used as matrix components, additives, and fillers in composites or hybrid composites [9,10]. Mixing polymers together and using plasticizers effectively improve the processability and strength of biopolymers. As a result, it increases the possibility of their potential applications in various branches of industry [11,12,13,14,15,16].

Given the current advances in biopolymer research, biopolymers from the poly(hydroxyalkaonates) (PHAs) family show properties with the most potential [17,18]. The physicochemical properties of short-chain PHAs are similar to polypropylene (PP) [19]. This, in turn, makes them predisposed to replace plastics made especially of PP in the future, especially disposable packaging.

**Figure 1:**
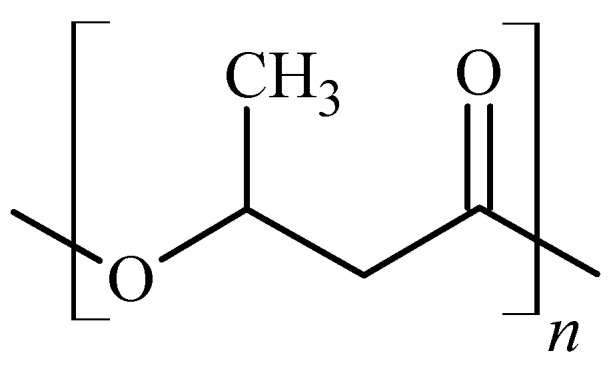
Repeated unit of P3HB.

A representative of the PHAs family is poly(3-hydroxybutyrate), (P3HB) (Figure 1), which is a common compound responsible for energy storage in bacteria. Due to its biocompatibility, non-toxicity, bioinertness, and biodegradability, it is used in many medical applications [20,21,22]. As a material, it has many disadvantages, most notably brittleness, low thermal stability, or a narrow processing window [23,24]. The degradation temperature of P3HB is almost equal to its melting point, which makes its processing conditions very difficult. Such disadvantages can be compensated for by certain modifications to P3HB depending on the selected application.

One of the methods to improve the properties of P3HB is to produce blends and composites using it as a polymer matrix. Taking into account the work of Bakar and co-workers on hybrid compositions and nanocomposites based on polyester resins with the addition of polyurethanes, an improvement in the thermal and mechanical properties of P3HB was expected after the introduction of polyurethane [25,26,27,28,29]. The use of a polyurethane modifier was not only supposed to improve the thermal and mechanical properties of P3HB but also should not reduce the biodegradability of P3HB because the addition of hydrophilic polymers increases the absorption of water into the polymer mass and accelerates its hydrolysis, facilitating biodegradation [30]. Therefore, various polyurethanes are used to modify the P3HB properties, i.e., polyurethanes based on hexamethylene 1,6-diisocyanate and polyethylene and polypropylene glycols with different molar masses were used to produce polymer compositions and composites [31,32,33,34].

The present studies aimed to modify the properties of P3HB by producing polymer blends with MDI-based linear polyurethanes and polyethylene glycols of different molar masses. The compatibility and homogeneity of the obtained polymer compositions were confirmed, and their thermal and mechanical properties were studied and compared with those of native P3HB.

## 2. Materials and Methods

### 2.1. Materials

P3HB was supplied by Biomer (Krailling, Germany). Its weight average molecular mass was M_w_ = 443,900 g·mol^−1^, and its dispersity index was (M_w_·M_n_^−1^) = 5.72. The P3HB melt flow index was 0.11 g·(10 min)^−1^ (180 °C at 2.16 kg). 4,4′-diphenylmethane diisocyanate (MDI) and dibutyltin dilaurate (DBTL) were supplied by Sigma-Aldrich (Saint Louis, MO, USA) and acetone from Chemsolute (Stuttgart, Germany).

### 2.2. Synthesis of Linear Polyurethanes with Aromatic Rings

In a three-neck round-bottom flask equipped with a mechanical stirrer, thermometer, and dropping funnel, polyethylene glycol with molecular mass 1000 g/mol (PEG1000), anhydrous acetone, and dibutyltin(IV) dilaurate (DBTL) as a catalyst (0.003 mol/mol PEG1000) were placed. The reaction was conducted in an inert gas (nitrogen). A suspension of 4,4′-diphenylmethane diisocyanate (MDI) in acetone was dropped into the solution in such a way that the molar ratio of isocyanate groups to hydroxyl groups of glycol was 1:1.08. The dropping funnel was protected by a tube with drying media. The dropping of the suspension was carried out at such a rate that the reaction mixture’s temperature did not exceed 30 °C. If the exothermic effect disappeared, the reaction mixture was kept boiling at about 56 °C for at least 1 h. The turbidity of the reaction mixture occurred during heating. The end of synthesis was confirmed by an increase in the viscosity of the mixture, as well as by a zero value of the determined isocyanate number (according to the standard PN-EN 1242, 2006 [35]). Acetone was removed from the reaction mixture using a rotary evaporator. Residual solvent was removed during the exposure of the polyurethane in a vacuum dryer at 40–100 °C to a constant product weight.

**Figure 2:**
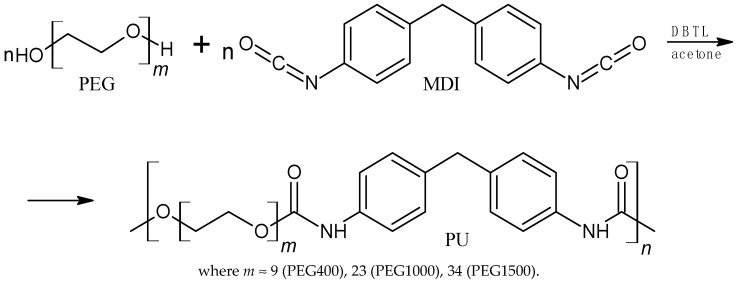
Synthesis scheme of polyurethanes.

Syntheses were also conducted using polyethylene glycol with a molar mass of 400 g/mol (PEG400) and a molar mass of 1500 g/mol (PEG1500) using the appropriate amount of catalyst, i.e., 0.001 mol DBTL/mol PEG400 and 0.005 mol DBTL/mol PEG1500 (Figure 2).

The polyurethanes were characterized by size-exclusion chromatography (SEC) by determining the number of the average molar mass (M_n_), the weight of the average molar mass (M_w_), the molar mass at peak maximum (M_p_), and the degree of dispersion (DI). The results of the SEC analysis are shown in Table 1 below.

### 2.3. Preparation of Polymer Blends

Polymer compositions consisting of poly(3-hydroxybutyrate) (P3HB) and a modifier, in the form of an aromatic linear polyurethane, were produced with PU contents of 5, 10, and 15 wt.% (Table 2). For this purpose, the polyurethane was heated in a laboratory dryer to a liquid state at 100 °C. In the next step, polyurethane was homogenized with P3HB by mixing them in a drum mixer. The homogenization process was carried out for 20 min at room temperature. The homogenized mixture was fed into a co-rotating twin-screw extruder, where the extrusion of biodegradable polymer blends was carried out. The different zones of the extruder were maintained at the following temperatures: tray—25 °C, zone I—125 °C, zone II—136 °C, zone III—150 °C, zone IV—160 °C, and head—166 °C. Extrusion was carried out at a speed of 350 rotations per minute. The molten product exited the extruder through a dual-core head. After cooling in a cooling bath, the composition was pelletized and then dried at 60 °C for more than 2 h.

### 2.4. Analytical Methods

#### 2.4.1. Size-Exclusive Chromatography (SEC)

The molar mass of the resulting polyurethane was measured by SEC using the following apparatus and measurement conditions: Waters HPLC liquid chromatograph, Waters model e2695 and Waters model 2414 differential refractometer, mixed gel bed column series: PL gel MIXED-A (300 × 7.5 mm, 20 μm) + PL gel MIXED-B (300 × 7.5 mm, 10 μm) + PL gel MIXED-D (300 × 7.5 mm, 5 μm), mobile phase: tetrahydrofuran (THF) stabilized with butylated hydroxytoluene, temperature: 40 °C, injection volume: 100 μL, flow rate: 1 mL/min, refractometer detector, and Empower 3 software.

Prior to the measurements, the chromatographic system was calibrated with polystyrene standards for molecular weights in the range of 580–990,500 g·mol^−1^ with 12 points.

#### 2.4.2. Scanning Electron Microscopy

The morphology of the polymer compositions was studied using a JEOL JSM-6490 LVy scanning electron microscope (Tokyo, Japan) with 20 kV accelerating voltage and secondary electron detection (SEI). The samples to be tested were frozen in liquid nitrogen and then shattered with a hammer. The surfaces of the shattered samples were then sputtered with a thin layer of gold about 10 nm thick using a JEOL JFC-1300 atomizer (Tokyo, Japan). Finally, the sputtered samples were placed in an electron-microscope chamber, and the material surface was analyzed in several micro-areas.

#### 2.4.3. Thermogravimetric Analysis

Thermogravimetric analysis (TGA) of the polymer compositions was carried out using a Metler Toledo TGA/DSC 3+ thermogravimetric analyzer (Hamburg, Germany). Composition samples were heated at a rate of 5 °C/min in the temperature range from +25 to +600 °C in an inert gas (nitrogen) atmosphere. The temperature of the onset decomposition (T_on_), the temperature corresponding to 50% weight loss (T_50%_), the temperature of the maximum decomposition rate (T_max_), and the total weight loss of the samples at 600 °C were determined.

#### 2.4.4. Standard Differential Scanning Calorimetry Measurements

The DSC measurements of P3HB, polyurethanes, and their biocompositions were carried out with the use of a differential scanning calorimeter (TA Instrument Q25, Newcastle, DE, USA). The results were obtained in the form of a heat-flow dependence on temperature or time as a response to a linear change of temperature with time. All measurements were made under a nitrogen atmosphere at a constant nitrogen flow of about 50 mL/min. The temperature and heat-flow calibration of in calorimeters was carried out in relation to the melting parameters of indium. The initial melting point, the so-called “Onset” of indium is T_m(onset)_ = 156.6 °C (429.6 K), and the enthalpy of fusion is ΔH_f_ = 28.45 J/g (3.28 kJ/mol).

The weights of the tested samples were in the range of 5–10 mg. The accuracy of measurements was ±3%. DSC measurements were carried out in the temperature range of −40 to 195 °C, and a constant heating rate of 10 °C·min^−1^. An isothermal annealing was performed for 2 min at 195 °C, and the system was stabilized for 5 min at −40 °C.

#### 2.4.5. FTIR Spectroscopy

The infrared (FTIR) spectra of P3HB, polyurethane, and their compositions were recorded on an ALPHA FT-IR Bruker instrument in ATR mode or KBr discs at the wavenumber of 400–4000 cm^−1^. The spectra were recorded at a resolution of 2 cm^−1^ using the ATR technique.

#### 2.4.6. Mechanical Properties

The following tests were carried out for the mechanical properties of the obtained blends and pure P3HB, i.e., tensile strength, relative elongation at break, Charpy impact strength, and shore hardness.

The tensile mechanical properties were determined in accordance with PN-EN ISO 527-2: 2012 [36] using the Instron 4505 testing machine. Tensile strength and relative elongation at break were measured at a tensile rate of 5 mm/min.

Charpy impact tests were conducted in accordance with PN-EN ISO 179-1: 2010 [37] using a Zwick 5102 impact hammer.

The hardness of the obtained polymer composition samples was determined by the shore method using Bariess Shore hardness testers: HP-A and HP-D. The measurement consisted of measuring the resistance of the sample of the tested composite during the penetration of a needle of a defined shape and dimension in accordance with PN-EN ISO 868 [38] and ASTM D2240 [39].

## 3. Results and Discussion

P3HB is a biodegradable, biocompatible, and biosynthesizable polymer [40]. However, P3HB exhibits undesirable characteristics, which include brittleness, stiffness, and limited thermal stability, making this polymer difficult to process [41]. P3HB was, thus, mixed with linear aromatic polyurethane in order to improve its selected properties. The linear polyurethane was prepared by the polyaddition reaction of diphenylmethane 4,4′-diisocyanate (MDI) and polyethylene glycol with a molar mass of 400 g/mol (PEG400), 1000 g/mol (PEG1000), or 1500 g/mol (PEG1500) in the presence of dibutyltin(IV) dilaurate (DBTL) as a catalyst.

Polyurethanes were used in amounts of 5, 10, and 15 wt.% (Table 2) to synthesize biodegradable polymer compositions. The blends were prepared using a co-rotating twin-screw extruder. P3HB processed under similar conditions as the polymer blends was used as the reference material for comparison purposes.

### 3.1. Spectral Analysis of the Prepared Polymer Biocompositions

**Figure 3:**
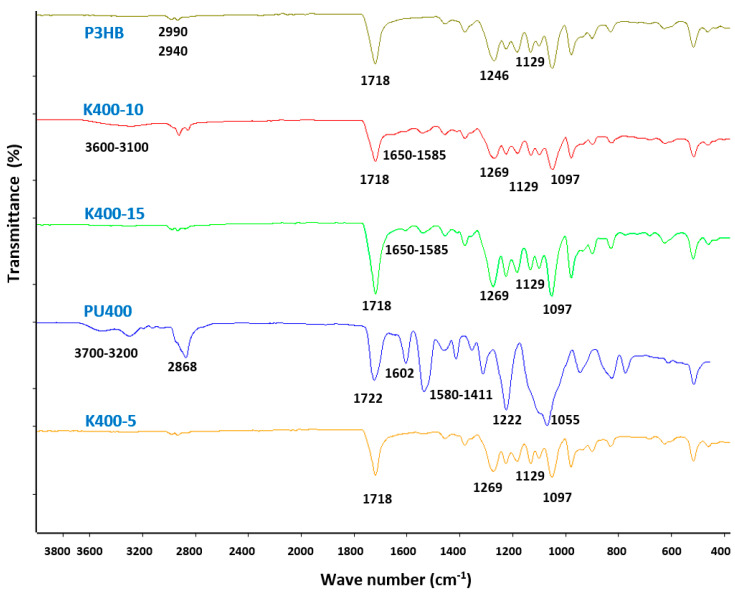
Set of FTIR spectra of native P3HB, PU400, and P3HB-PU polymer biocompositions containing 5, 10, and 15 wt.% PU400 (K400-5, K400-10, and K400-15, respectively).

FTIR characterization was carried out aiming to verify the possible interactions between the components of the biocompositions and their compatibility [42]. The spectra of the prepared P3HB-PU compositions were compared with the FTIR spectra of their integral components, i.e., native P3HB as well as PU400, PU1000, and PU1500. The observed differences and similarities of the bands appearing in the FTIR spectra are discussed in the example of polymer compositions prepared from PU400 as presented in Figure 3.

All characteristic peaks were observed in the FTIR spectrum of native P3HB (Figure 3), thus confirming the structure of the biocomposition matrix used. The spectrum showed a characteristic, intense band of ester bonds, originating from the valence vibrations of C=O bonds at 1718 cm^−1^. Additionally, asymmetric and symmetric vibration bands of the C–O bonds of the ester group are observed at 1246 cm^−1^ and 1129 cm^−1^. However, asymmetric and symmetric vibration bands of C–H bonds in methyl and methylene groups are observed at 2990 cm^−1^ and 2940 cm^−1^.

On the FTIR spectrum of PU400 (Figure 3), there is a band from the valence vibration of the N-H bond of the urethane group –NH–COO– in the wavenumber range of 3600–3100 cm^−1^. The peak of the urethane groups is wide and diffuse, indicating the occurrence of hydrogen bonds in polyurethane. At 2868 cm^−1^, one broad band from valence vibrations of asymmetric and symmetric C–H bonds of methylene groups PU400 is also observed. At about 3060 cm^−1^, there is a band of C–H bond vibrations of the phenylene ring. A valence vibration band of the C=O groups of urethanes –NH–COO– appears at 1722 cm^−1^, and at 1602 cm^−1^, a band characteristic of deformation vibrations of N–H bonds of polyurethane appears. In the wavenumber range of 1580–1411 cm^−1^, the characteristic backbone bands of the aromatic ring are present. Bands of asymmetric and symmetric C–O bonds of –NH–COO– urethane groups are visible at 1222 cm^−1^ and 1055 cm^−1^. The FTIR analysis of the polyurethane used to modify P3HB, therefore, confirmed the correct progress of the polyaddition reaction during PU synthesis.

Similarities and differences were found between the spectra of biocompositions containing different amounts of PU (Figure 3). These spectra also differ in their intensity and range of peaks compared to the peaks appearing in the spectrum of the native polymer matrix and the polyurethane modifier itself. In the spectra of all P3HB-PU biocompositions (Figure 3), there is, in the range of 2800–3000 cm^−1^, a scattered band with variable intensity depending on the amount of PU400 in the composition. In the biocomposition containing 10 wt.% PU, this band is much smaller than in native polyurethane but larger than in P3HB itself. This may indicate that the urethane groups of the polymeric modifier interreacted with the CH and CH_2_ groups of P3HB in the composition containing 10 wt.% PU. However, in the samples containing 5 wt.% or 15 wt.% polyurethane, the intensity of this band is comparable to the band assigned to the vibrations of C–H methine and methylene groups in P3HB, which may confirm the lack of interaction of the biomatrix with such amounts of polymer modifier. Moreover, there is a band at the wave number of 1718 cm^−1^ in all the spectra of the biocompositions. It was attributed to the stretching vibrations of C=O bonds of the –NH–COO– urethane group of the PU modifier. Additionally, it should be emphasized that the lowest intensity of this band was shown by the biocomposition containing 10 wt.% PU (K400-10), demonstrating the occurrence of the urethane groups’ interaction in the biocomposition components. In the wave number range of 1585–1650 cm^−1^, there is a band of bending vibrations of the N-H bonds of urethane, which is characterized by multiplicity due to the formation of intermolecular hydrogen bonds between the P3HB and PU400 chains [43,44]. The intensity of this band is variable and depends on the amount of PU400 in the polymer composition and is highest for a 10 wt.% amount of PU. Hence, it can be assumed that the interactions between the matrix and P3HB occurred to the greatest extent in the sample modified with 10 wt.% PU. Additionally, other bands characteristic of the analyzed compositions were identified. Common bands from asymmetric and symmetric C–O vibrations of esters and urethanes appeared at a wavenumber of 1269 cm^−1^ and 1129 and 1097 cm^−1^.

An FTIR spectral analysis of the prepared polymer compositions and their components confirmed the interaction of polyurethane with the P3HB matrix in a composition containing 10 wt.% polymeric modifier.

In the spectra of biocompositions obtained with other polyurethanes, i.e., PU1000 and PU1500, similar changes are observed with a change in the amount of PU in the polymer biocompositions.

### 3.2. Morphology Characteristics of the Obtained Polymer Biocompositions

**Figure 4:**
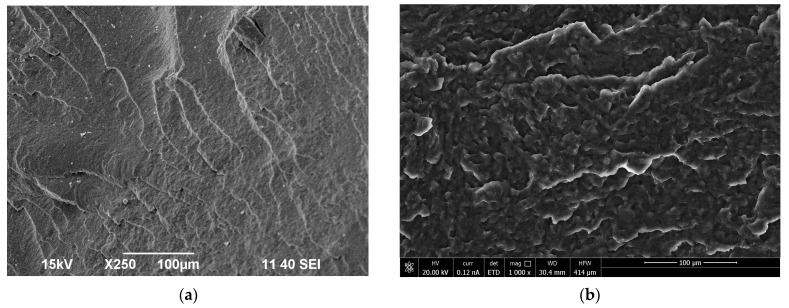

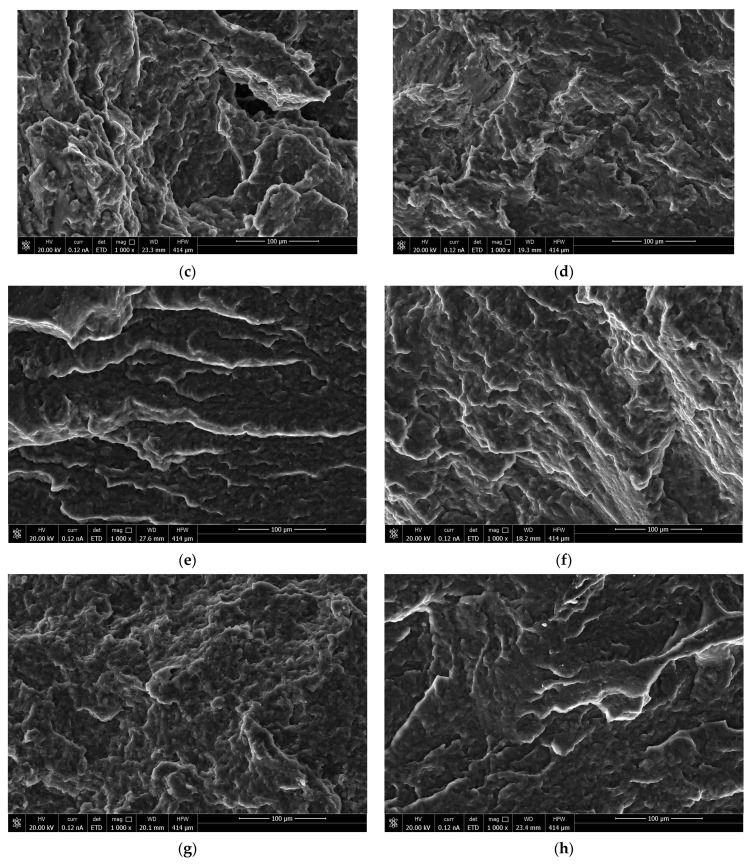

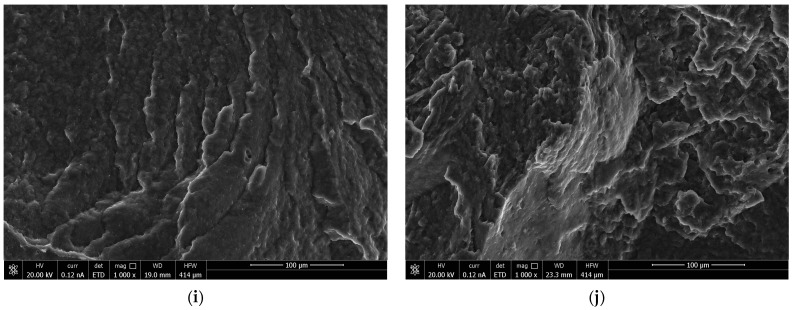
SEM micrographs of P3HB (**a**) and its polymer biocompositions containing 5, 10, and 15 wt.% polyurethane: PU400—K400-5 (**b**), K400-10 (**c**), K400-15 (**d**); PU1000—K1000-5 (**e**), K1000-10 (**f**), K1000-15 (**g**); PU1500—K1500-5 (**h**), K1500-10 (**i**), and K1500-15 (**j**).

Figure 4 shows SEM micrographs of the fracture surfaces of polyester samples and biocompositions containing 5, 10, and 15 wt.% of polyurethane modifier based on polyethylene glycol (PEG400, PEG1000, and PEG1500). The fracture surfaces of the broken samples are used, in general, to explain the possible interaction between the polymer matrix and the polyurethane modifier and, hence, the mechanism(s) that may explain the improvement in the mechanical properties of the tested biocompositions. The images presented were obtained by scanning the surfaces of broken samples. The fracture surface of unmodified polyester (P3HB), shown in Figure 4a, is slightly wavy and glassy, indicating a regular and relatively linear crack propagation path. In addition, the brittle cracking zones are arranged unidirectionally [45].

Figure 4b–j show the fracture surfaces of P3HB polyester biocompositions modified with different amounts of polyurethane based on short-chain-based glycol (PEG400) and long-chain glycols (PEG1000) and (PEG1500). As can be seen, the introduction of the PU modifier caused an apparent disruption in the continuity of the P3HB matrix structure. The rough areas visible in the images and arranged in different directions may suggest the interaction of the biopolymer matrix with PU, leading to the separation of the interacting P3HB chains and the formation of the mentioned domains.

There were clear differences in the aforementioned disentanglement when comparing the images of PU400-modified samples with the structure of the PU1000- and PU15000-modified samples, and it was greater the longer the chain of glycol was used (Figure 4b–j). Such a relationship may be related, for example, to an increase in the elongation at break, to a slight decrease in hardness and tensile strength, and to an increase in the impact strength of compositions based on long-chain PU compared to samples modified with short-chain PU400 [46]. Thus, slight differences in the mechanical properties of the samples based on PU1000 and PU1500 are expected.

The composition structure was also affected by the amount of added PU. The polyester matrix modified with 10 wt.% PU had a slightly different structure than the other samples (compositions containing 5 wt.% or 15 wt.% PU. The distinct rough zones and those unidirectionally aligned are much larger in size than in the other images of compositions containing 5 wt.% and 15 wt.% PU. Moreover, the unidirectionally arranged rough zones are longer the longer the PU chains are. This finding demonstrated that the higher the molecular weight of PEG used for PU synthesis, the better the plasticization effect of P3HB. This may be related to the formation of P3HB-PU adducts that are easily displaced relative to each other. The images of compositions with PU1000 and PU1500 are similar at 15 wt.% PU, which may account for the slight increase in elongation at break and little or no difference in impact strength of the analyzed samples. On the other hand, the use of PU1500 resulted in the formation of PU agglomerates in the matrix, with the deterioration of the mechanical properties compared to the PU1000-modified compositions.

### 3.3. Mechanical Properties of the Produced Polymer Biocompositions

**Figure 5:**
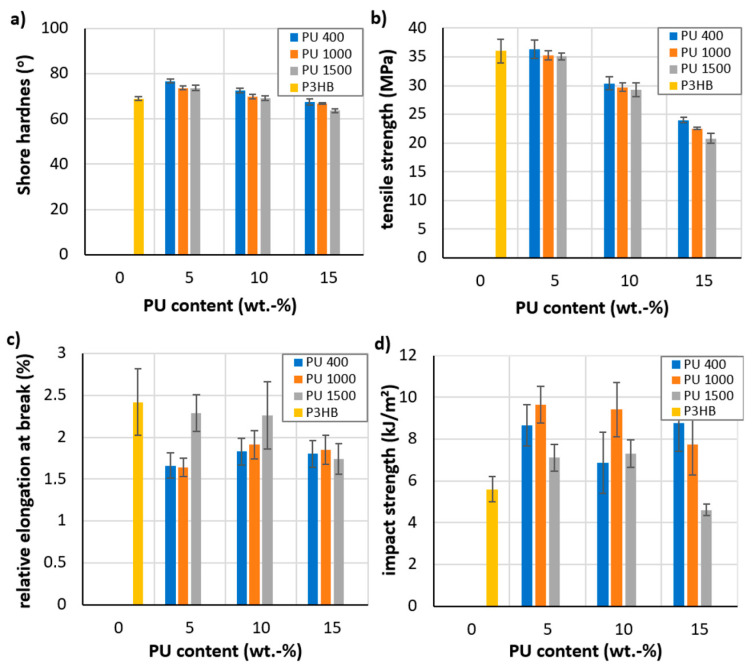
Effect of PU400, PU1000, and PU1500 content on hardness (**a**), tensile strength (**b**), relative elongation at break (**c**), and impact strength (**d**) of P3HB-PU polymer biocompositions.

Selected mechanical properties of the prepared polymer compositions were evaluated, and the obtained results are shown in Figure 5. Figure 5a shows an increase in the hardness of obtained polymer biocompositions based on P3HB-PU. The hardness of polymer compositions with PU400 decreased with an increase in the amount of added PU, but it was still higher than that of the unmodified P3HB. Similar results were observed in the compositions with PU1000. Furthermore, it is shown that even the modifier PU1500 caused the decrease of hardness below the hardness value of native P3HB. It was observed that after an initial increase of hardness the polymer biocompositions compared to native P3HB, that the longer the chain of polyethylene glycol used to prepare the polyurethane, the greater the decrease in hardness, i.e., the greater the flexibilization effect of the polyurethane modifier, despite the presence of the aromatic rings [47].

The addition of PU based on MDI to P3HB causes two effects, namely the flexibilization effect of the polyol chain and the stiffening effect of aromatic rings. The stiffening effect of aromatic rings predominates when the polyol chains are shorter (PU400 and PU1000). In the case of PU1500, the flexibilization effect of the polyol chain starts to prevail.

Figure 5b shows the dependence of tensile strength (TS) on the content of PU400, PU1000, and PU1500 in the polymer biocompositions, and indicates that the strength decreased with the increasing molecular weight of polyethylene glycol in PU [48]. The TSs of all the P3HB-PU polymer compositions were lower than that of unmodified P3HB, apart from the TS of the 5 wt.% PU400-based composition, which was slightly higher. An increase in the amount of PU in the polymer composition from 5 wt.% to 10 or 15 wt.% resulted in a minimal decrease in the TS values. The introduction of PU1000 (wt.5%) into P3HB resulted in only a minimal decrease in the tensile strength of the polymer biocomposition. However, increasing the amount of PU1000 up to 10 wt.% resulted in an approximately 16% decrease in the TS. The lowest value of TS (i.e., a decrease in strength of more than 30% compared to a native P3HB) was shown by the P3HB-PU polymer biocompositions with PU1500 (wt.15%). It should be noted that, in the case of modification of P3HB with PU1500, an increase in the amount of PU caused a regular decrease in the TS of the biocompositions. The reduction in TS can be explained by the flexibilization of the biocompositions induced by the flexible chains of the polymer modifier, as demonstrated by the hardness results (Figure 5a).

In the case of the relative elongation at break (Figure 5c), it can be noted that the modification of P3HB with the linear aromatic polyurethanes PU400, PU1000, and PU1500 led to the decrease in the value of the parameter of all the tested compositions. Strength–strain curves are shown in Figures S1–S4. The lowest value of the relative elongation at break was shown by the blends containing the PU400 modifier. An increase in the PU400 content results in a small increase in elongation at the break. A similar relationship is observed in the case of the introduction of PU1000. Attention is focused on the polymer composition containing 5 and 10 wt.% PU1500, where a significant increase in the relative elongation at break is observed. In the case of the polymer composition containing 15 wt.% PU1500, the relative elongation at the break parameter showed lower values. Another study has confirmed that the addition of small amounts of vegetable-oil-derived plasticizers resulted in an increase in elongation at break, with a significant increase in the impact strength [49,50]. Moreover, the use of terpene plasticizers led to a significant increase in elongation at the break (more than a six-fold increase of the P3HB, which increases free volume and molecular mobility) [51].

The impact strength (IS) of the P3HB-PU polymer compositions is shown in Figure 5d as a function of the PU modifier content. It should be noted that, basically, all polymer biocompositions showed higher IS than the native P3HB sample. The maximum IS (70% improvement over native P3HB) was shown by the polymer compositions containing 5 and 15 wt.% PU400, as well as 5 and 10 wt.% PU1000, which is in accordance with the results obtained by SEM. Among the compositions with PU1000, the lowest IS was found in the composition containing 15 wt.% PU1000. On the other hand, the impact resistance increased and then decreased after the addition of PU1500. At 15 wt.% PU1500, the composition showed the smallest IS value, being the only one even smaller than that of the native P3HB (the IS reduction reached ~17%). The flexibilizing effect induced by the longest polyol chain in the polyurethane based on PU1500 was reflected by the lowest hardness and, at the same time, the lower IS of the P3HB-PU samples [52].

### 3.4. Thermal Stability of the Prepared P3HB Polymer Blends

The thermal stability of the prepared polymer biocompositions and native P3HB was determined by thermogravimetric (TG) analysis. The TG results are shown in Table 3. Taking into account that P3HB can partially degrade in the extruder as Yeo et al. [20] described, the native P3HB was processing in the same or very similar conditions as the P3HB-PU compositions.

The thermal stability of the P3HB-PU polymer biocompositions, as measured by the onset decomposition temperature (Ton), increased by 30 °C compared to pure P3HB [53,54]. As it has been reported by Garcia-Garcia et al. [50], the thermal stability of P3HB increased by more than 30 °C due to the addition of a plasticizer. By onset decomposition temperature, we mean the temperature at which a detectable weight loss of less than 5% occurs. Moreover, it can be noted that the amount and type of incorporated PU, basically, did not affect the values of T_on_. Due to the specificity of the P3HB-based composition, the thermal decomposition occurred in one step, with a maximum in the temperature range of 283–287 °C, as observed in the DTG curve, further indicating the fastest decomposition of the material. The maximum decomposition temperature of the native P3HB was close to the temperatures of all the prepared P3HB-based compositions. The residue after decomposition at 600 °C was similar, not exceeding 2 wt.%, and was slightly higher than that of unmodified P3HB, which is related to the presence of aromatic structures introduced into the biocompositions with polyurethanes. The preparation of P3HB polymer compositions with aromatic linear polyurethanes contributed to an increase in the thermal stability of the obtained materials.

### 3.5. Analysis of Thermal Parameters of P3HB-PU Polymer Biocompositions

**Figure 6:**
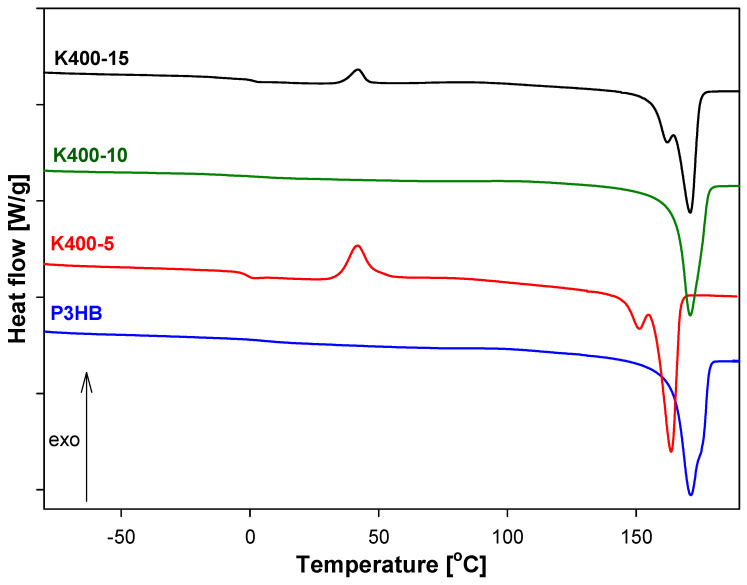
Comparison of heat flow of poly(3-hydroxybutyrate), P3HB, and K400-5, K400-10, K400-15 biocompositions versus temperature.

**Figure 7:**
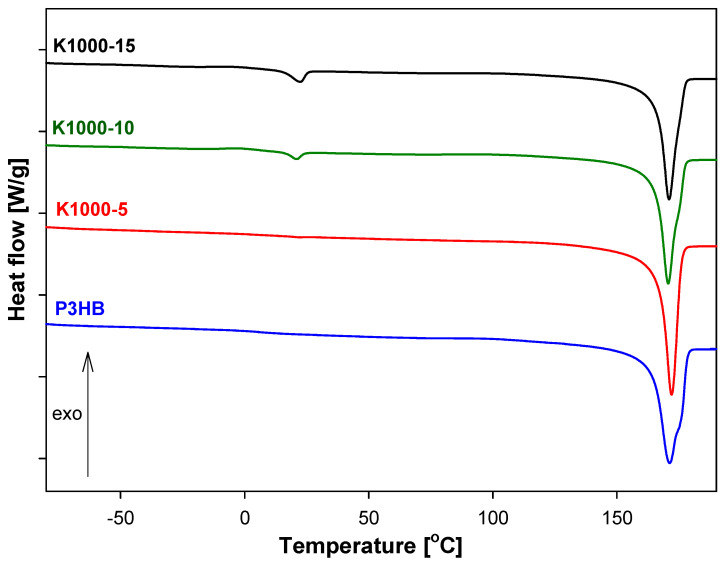
Comparison of heat flow of poly(3-hydroxybutyrate), P3HB, and K1000-5, K1000-10, K1000-15 biocompositions versus temperature.

**Figure 8:**
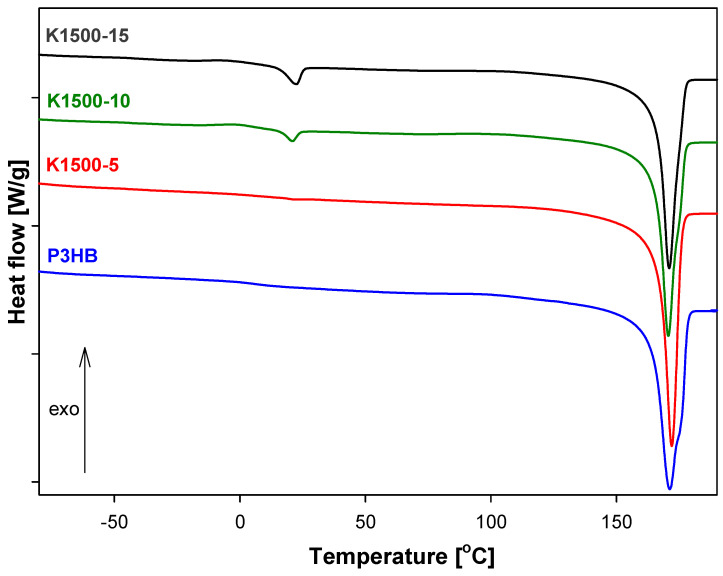
Comparison of heat flow of poly(3-hydroxybutyrate), P3HB, and K1500-5, K1500-10, K1500-15 biocompositions versus temperature.

Figure 6, Figure 7 and Figure 8 show the heat-flow rates versus the temperature in the range of −90 °C to 195 °C for K400-, K1000-, and K1500-based biocompositions. All obtained data are presented in Table 4. The qualitative thermal analysis was performed based on the heat-flow rates of semicrystalline P3HB and its blends with PU and recording glass transition (T_g_), cold crystallization, and melting temperatures. Based on the analysis of the glass transition region during heating, the change of heat capacity (ΔC_p_) and the value of T_g_ were determined. However, from the analysis of the melting transition, the heat of fusion (ΔH_f1_ and ΔH_f2_) and the beginning of melting T_m1(onset)_ and T_m2(onset)_ were estimated.

Figure 6 shows three heating scans for K400 blends and P3HB as the reference sample. The glass transition, cold crystallization, and melting were observed for K400-5 and K400-15. Probably, it is linked to the method of extrusion of the blends and the forming of some of the contents of the crystalline phase during heating. It can be observed that glass transition and melting are visible for K400-10. The glass transitions of K400-5 and K400-15 were observed in the range of −1.80 °C to 1.20 °C, and the onset melting temperature was in the range of 156.6 °C–163.2 °C. The change in heat capacity and heat of fusion were also determined for the next estimation of the phase content of new materials. Based on Table 4, the lowest value of the glass transition temperature was observed for the K400-5 biocompositions, which allows us to assume that this material will be the most plasticized compared to the other tested materials. The greatest processing capabilities K400-5 offers are because the processing window was estimated as 95.5 °C. For the remaining biocompositions (i.e., K400-10 and K 400-15), it is 87.4 °C and 90.4 °C, respectively.

**Figure 9:**
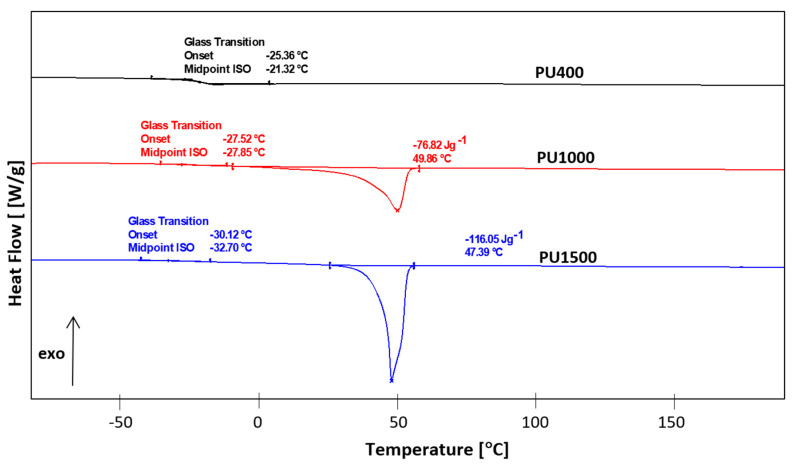
Comparison of heat flow of the used polyurethanes, PU400, PU100, and PU1500 versus temperature.

Figure 7 and Figure 8 show a single elongated glass transition and two melting peaks, which were caused by P3HB and PU contents. Figure 9 shows the melting peaks of PU1000 and PU1500, with the peak temperatures of melting at 49.86 and 47.39 °C, respectively.

The glass transition temperature of the polymers PU400, PU1000, and PU1500 was also determined. The glass transition temperature values of these polymers decrease in the series PU400→PU1000→PU1500, from −21.3 to −32.7 °C.

The glass transitions of K1000 and K1500 were observed in the range of −39.80 °C to −43.75 °C and 1.15 °C to 3.8 °C, respectively. Two melting peaks were observed in thermograms (Figure 7 and Figure 8) of all the biocompositions. The first, always smaller, endothermic peak is due to the melting of less stable crystals of PU (T_m1(peak)_, Table 4), while the second larger peak (T_m2(peak)_, Table 4) corresponds to the melting of larger, well-formed, crystals of P3HB. This same situation is observed for K1500 biocompositions. Based on Table 4, the lowest value of the glass transition temperature was observed for the K1000-10 biocomposite, which allows us to assume that this material will be the most plasticized in reference to the series of materials considered. The greatest processing capabilities K1500-15 offers are because the processing window was estimated as 87.2 °C. For the remaining biocompositions, K1000-5, K1000-10, K1000-15, K1500-5, and K1500-10 are 83.15 °C, 86.10 °C, 84.79 °C, 86.70 °C, and 85.9 °C, respectively. The remaining compositions from the K1000 and K1500 series improve also the P3HB processing window, but it is smaller. In the results, the best thermal properties have K400-5 and K1000-10.

All compositions have only one glass transition. In the case of compositions K400 and K1500, the value of the glass transition temperature is close to the Tg of P3HB and was shifted to lower temperatures. It is caused by the polymer interactions in the form of hydrogen bonds, the formation of which was confirmed by FTIR analysis. Compositions K1000 have a Tg value close to the Tg value of PU1000 and even lower than it. The coexistence of two melting peaks of compositions P3HB with PU1000 and PU1500 indicates a two-phase system and the formation of the interpenetrating polymer networks. In turn, the compositions of P3HB and PU400 are one-phase systems and are mixtures of thermodynamically compatible polymers.

## 4. Conclusions

1. New polymer biocompositions were prepared with poly(3-hydroxybutyrate) and aromatic linear polyurethane obtained by reacting diphenylmethane 4,4′-diisocyanate with polyethylene glycol at molecular weights of 400, 1000, and 1500 g/mol to produce the polymer compositions with polyurethane used in amounts of 5, 10, and 15 wt.%;
2. Spectral analysis (FTIR) confirmed the interactions of polymers with hydrogen bonds and their compatibility in the preparation of polymer biocompositions;
3. SEM analysis demonstrated that the morphology of the obtained polymer compositions showed a homogeneous structure, and the interaction of biopolyester with polyurethanes with the formation of P3HB-PU adducts easily displaced relative to each other. It resulted in the effect of P3HB plasticization, and the effect was better when the longer chain of PEG in PU was there;
4. The plasticizing effect of PU resulted in better mechanical properties for the new biocompositions compared to native P3HB. The prepared polymer biocompositions were characterized by a significant desirable increase in impact strength, an acceptable decrease in tensile strength, and a relative elongation at break, which was related to the plasticizing effect of PU on the properties of P3HB. The best mechanical properties were characterized by biocompositions containing 5 and 10 wt.% polyurethane PU400 and PU1000 modifiers, as confirmed by the analysis of the morphology of these blends;
5. The prepared polymer biocompositions were characterized by higher thermal stability compared to unmodified P3HB. The onset decomposition temperature of the polymer biocompositions was higher by 30 °C on average. Increasing the amount of added polyurethane modifier did not significantly affect the thermal stability of the blends obtained, as the values of the onset decomposition temperature of all the compositions tested were similar. The compositions with PU400 and PU1000 additives had the highest thermal stability;
6. The study carried out showed that the preparation of P3HB polymer biocompositions with the modifier in the form of aromatic linear polyurethane had a positive effect on the thermal and mechanical properties and significantly increased the processability of this polyester. It will be used in the future application of the compositions, among others, in the production of non-woven fabric.

## Figures and Tables

**Table 1:** Molar masses and dispersion degree of the obtained polyurethanes.

Polyurethane	M_n_ (g/mol)	M_w_ (g/mol)	M_p_ (g/mol)	DI
PU400	5000	7000	9000	1.40
PU1000	6000	8000	9000	1.33
PU1500	6000	8000	10,000	1.33

**Table 2:** Composition of the polymer mixtures.

**PU400 Content (wt.%)**	**Biocomposition Designation**
5	K400-5
10	K400-10
15	K400-15
**PU1000 content (wt.%)**	-
5	K1000-5
10	K1000-10
15	K1000-15
**PU1500 content (wt.%)**	-
5	K1500-5
10	K1500-10
15	K1500-15

**Table 3:** Interpretation of TG curves of pure P3HB and its biocompositions with PU obtained at a heating rate of 5 °C/min in a nitrogen atmosphere.

Sample	T_on_ (°C)	T_5%_ (°C)	T_10%_ (°C)	T_50%_ (°C)	T_max_ (°C)	Residue at 600 °C (%)
P3HB	221.1	236.2	245.6	281.2	282.1	1.10
K400-5	252.1	266.5	271.1	307.6	283.7	1.23
K400-10	252.7	267.1	271.5	329.3	285.7	1.40
K400-15	253.6	267.3	271.8	283.8	285.2	1.90
K1000-5	250.1	265.0	270.2	284.5	285.1	1.67
K1000-10	251.7	265.6	271.0	285.7	287.1	1.60
K1000-15	250.9	267.6	272.2	282.6	286.2	1.76
K1500-5	250.2	265.5	267.3	281.3	286.1	1.57
K1500-10	249.8	267.4	271.4	282.7	287.2	1.68
K1500-15	250.8	266	270.5	282.7	282.6	1.78

**Table 4:** Comparison of thermal parameters of P3HB, biocompositions, and polyurethanes upon heating their samples at 10 °C/min after prior cooling at the same rate.

Sample	T_g_ (°C)	ΔC_p_ (J·g^−1^·°C^−1^)	T_m1(Onset)_ (°C)	T_m1(Peak)_ (°C)	ΔH_f1_ (J·g^−1^)	T_m2(Onset)_ (°C)	T_m2(Peak)_ (°C)	ΔH_f2_ (J·g^−1^)
P3HB	7.70	0.162	159.73	165.75	91.93	-	-	-
K400-5	−1.80	0.358	156.60	163.60	37.89	-	-	-
K400-10	0.40	0.226	165.30	170.90	87.33	-	-	-
K400-15	1.20	0.160	163.20	171.00	58.25	-	-	-
K1000-5	−40.74	0.076	14.80	21.80	0.535	166.95	172.00	92.68
K1000-10	−43.75	0.024	16.13	20.85	0.959	165.60	170.70	86.30
K1000-15	−39.80	0.098	15.32	22.30	5.029	166.11	171.03	81.27
K1500-5	3.80	0.168	34.10	39.00	0.388	163.50	169.60	88.20
K1500-10	3.50	0.112	33.10	41.20	4.833	163.90	171.20	86.68
K1500-15	1.15	0.137	35.80	42.12	8.427	163.60	169.60	84.68
PU400	−21.32	0.296	-	-	-	-	-	-
PU1000	−27.85	0.143	3.42	49.86	76.82	-	-	-
PU1500	−32.70	0.098	27.05	47.39	116.05	-	-	-

## Data Availability

The raw data supporting the conclusions of this article will be made available by the authors on request.

## Supplementary Materials

The following supporting information can be downloaded at https://www.mdpi.com/article/10.3390/polym16121618/s1: Figure S1: Strength-strain curves of P3HB-PU polymer biocompositions containing: 5, 10 and 15 wt.% polyurethane: PU400—K400-5, K400-10, K400-15; Figure S2: Strength-strain curves of P3HB-PU polymer biocompositions containing: 5, 10 and 15 wt.% polyurethane: PU1000—K1000-5, K1000-10, K1000-15; Figure S3: Strength-strain curves of P3HB-PU polymer biocompositions containing: 5, 10 and 15 wt.% polyurethane: PU1500—K1500-5, K1500-10, K1500-15; Figure S4. Strength-strain curves of P3HB.
