# Supplementary material for: Biopolymer Compositions Based on Poly(3-hydroxybutyrate) and Linear Polyurethanes with Aromatic Rings—Preparation and Properties Evaluation
Source: Polymers (Basel). 2024 Jun 7;16(12):1618. doi: 10.3390/polym16121618 (PMC11207839; doi:10.3390/polym16121618)
Supplement: Supplementary file 1 [file polymers-16-01618-s001.zip › polymers-2980633-supplementary.pdf]

Electronic Supporting Information (ESI)

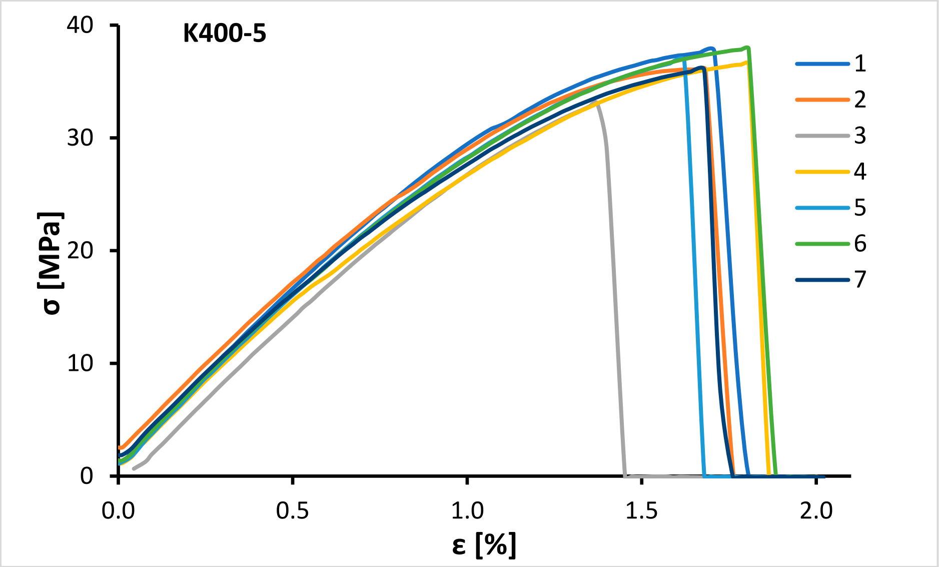

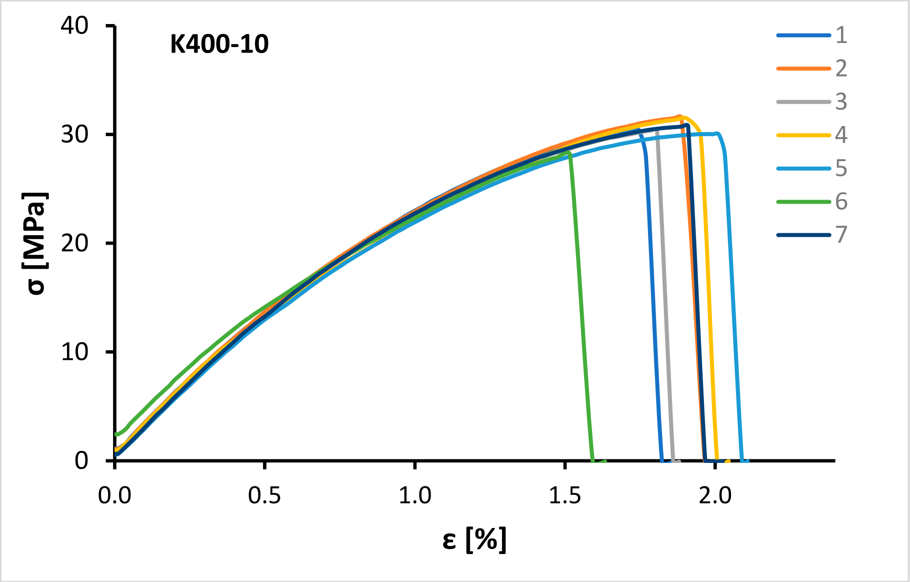

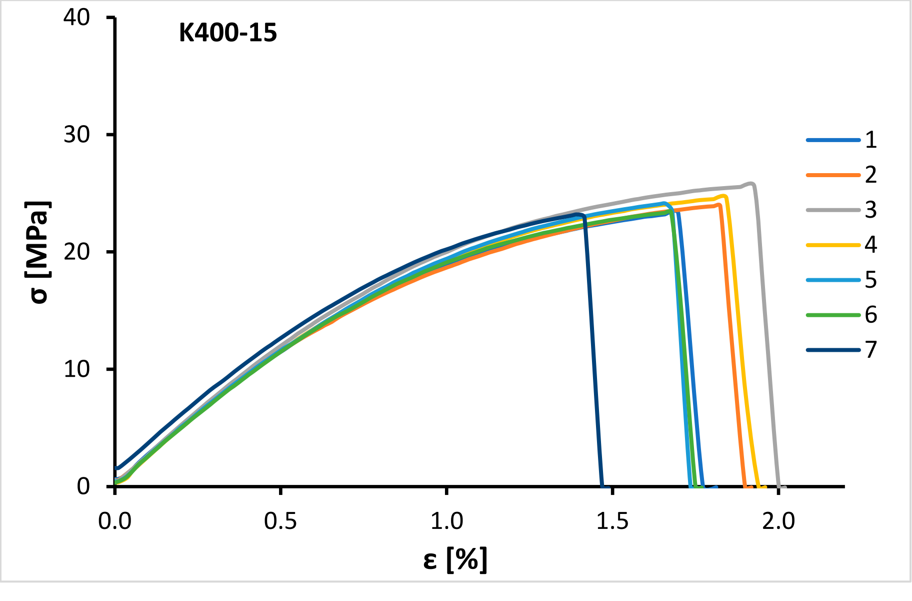

Figure S1. Strength-strain curves of P3HB-PU polymer biocompositions containing: 5, 10 and 15 wt.% polyurethane: PU400 – K400-5, K400-10, K400-15

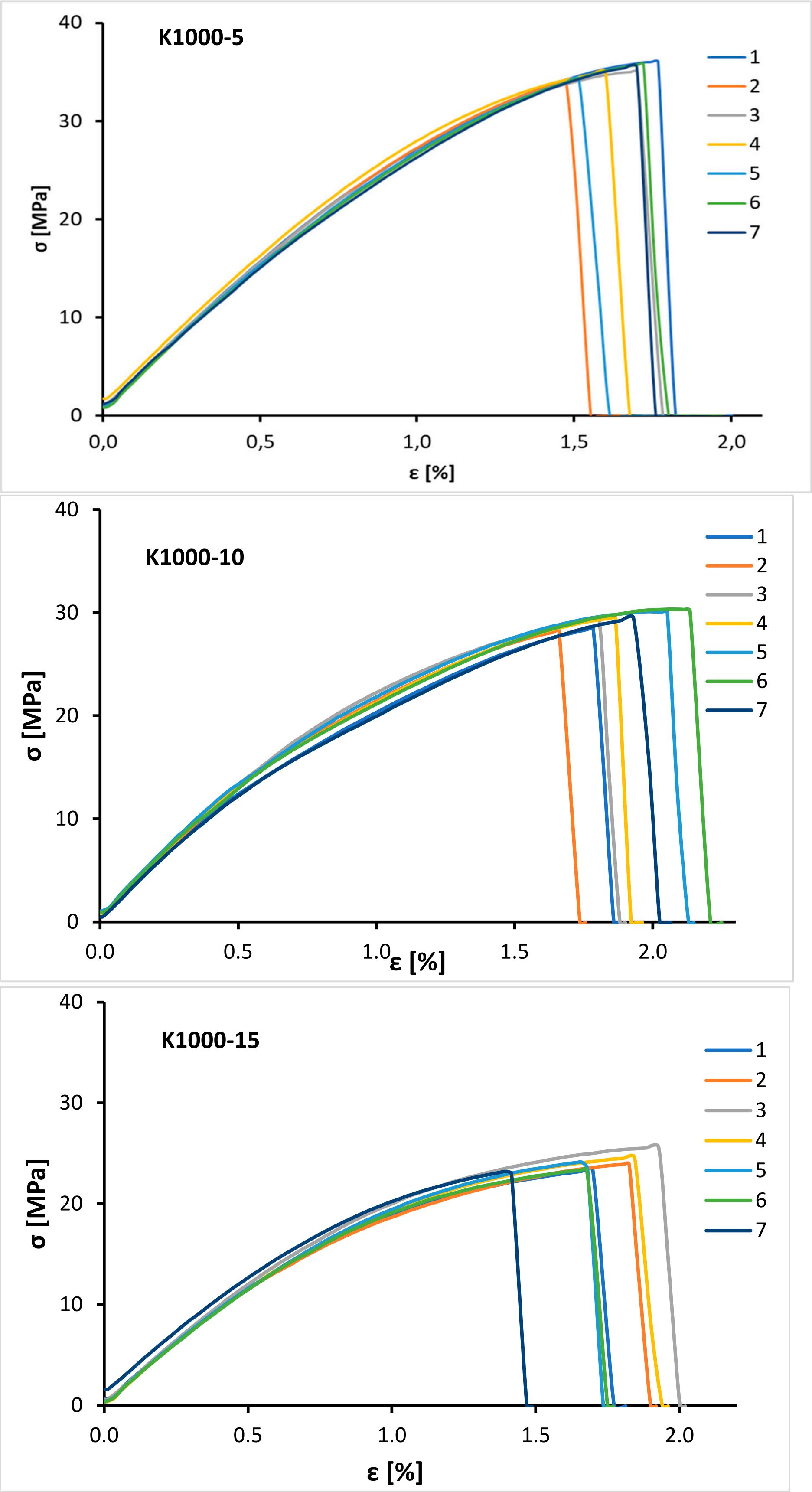

Figure S2. Strength-strain curves of P3HB-PU polymer biocompositions containing: 5, 10 and 15 wt.% polyurethane: PU1000 – K1000-5, K1000-10, K1000-15

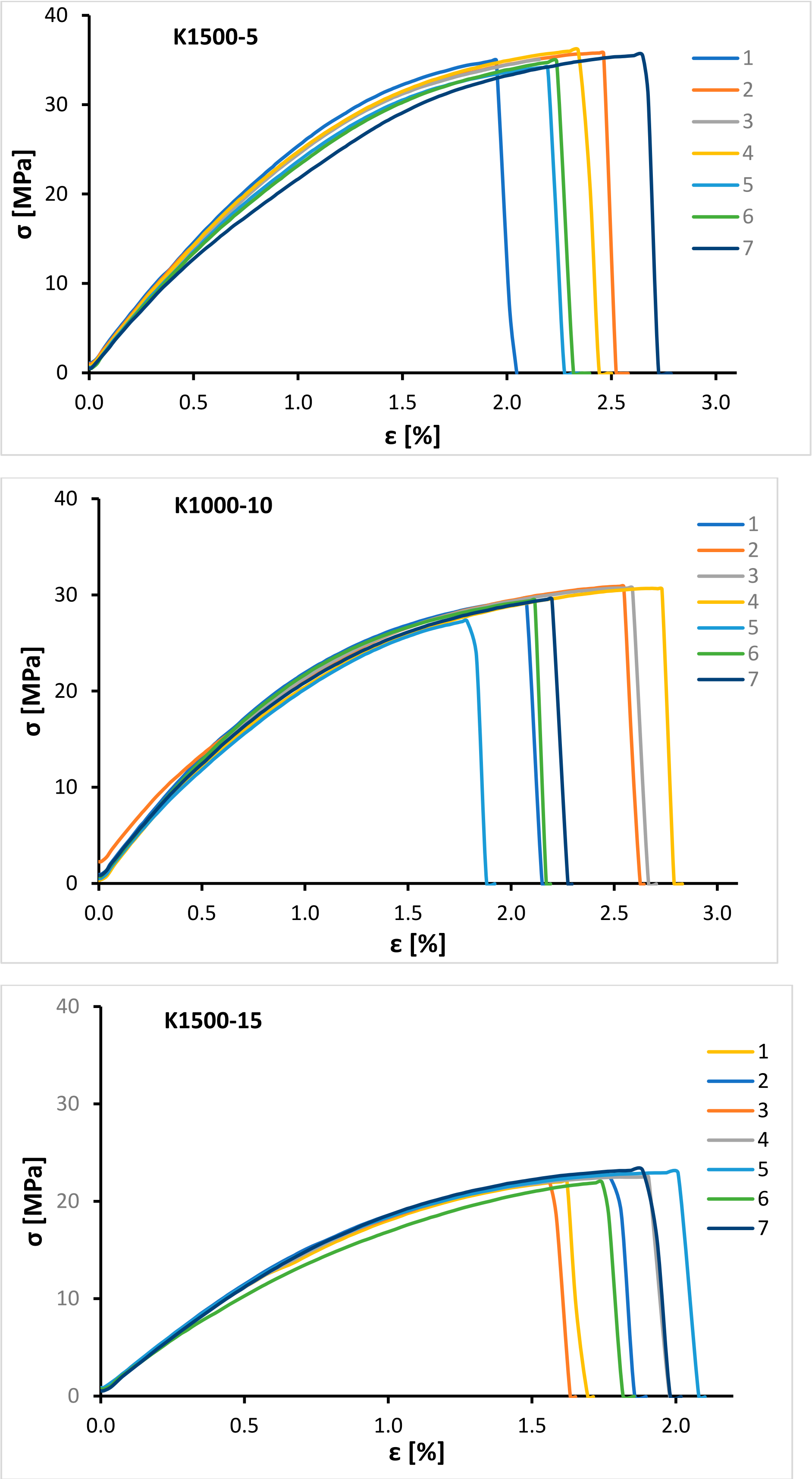

Figure S3. Strength-strain curves of P3HB-PU polymer biocompositions containing: 5, 10 and 15 wt.% polyurethane: PU1500 – K1500-5, K1500-10, K1500-15

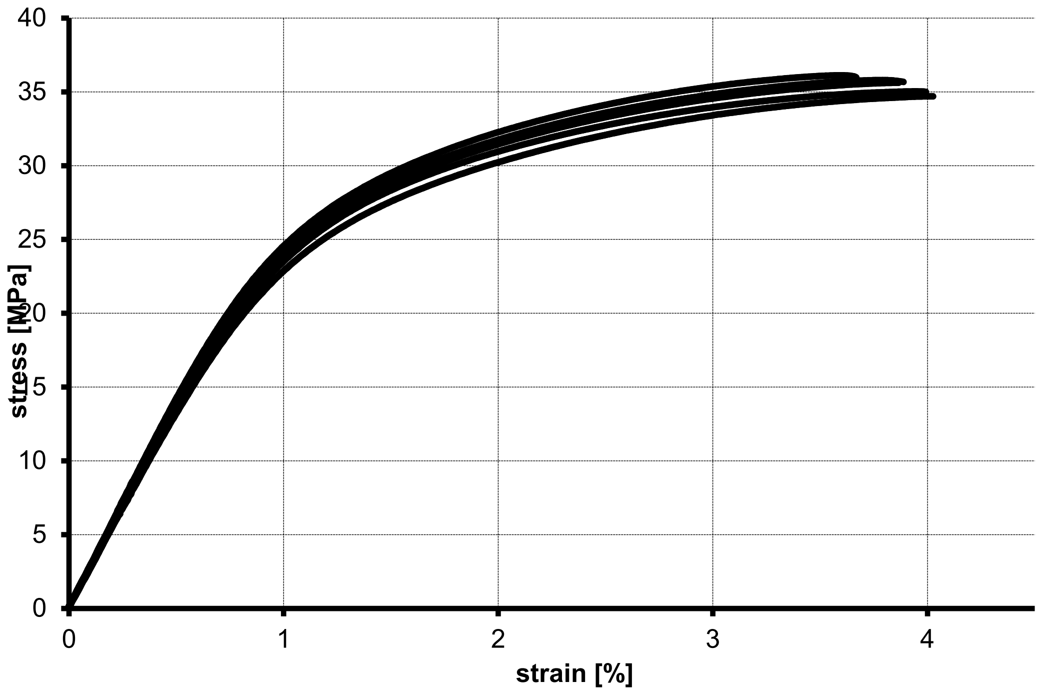

Figure S4. Strength-strain curves of P3HB
